# Evaluation of In-Ear and Fingertip-Based Photoplethysmography Sensors for Measuring Cardiac Vagal Tone Relevant Heart Rate Variability Parameters

**DOI:** 10.3390/s25051485

**Published:** 2025-02-28

**Authors:** Ankit Parikh, Gwyn Lewis, Hamid GholamHosseini, Usman Rashid, David Rice, Faisal Almesfer

**Affiliations:** 1 School of Clinical Sciences, Auckland University of Technology, Auckland 0627, New Zealand; 2 Exsurgo Ltd., 45i William Pickering Drive, Rosedale, Auckland 0632, New Zealand; 3 Department of Physiotherapy, Auckland University of Technology, Auckland 0627, New Zealand; 4 Department of Electrical and Electronic Engineering, Auckland University of Technology, Auckland 1010, New Zealand; 5 Centre for Chiropractic Research, New Zealand College of Chiropractic, Auckland 1060, New Zealand; 6 Health and Rehabilitation Research Institute, Auckland University of Technology, Auckland 0627, New Zealand; 7 Waitemata Pain Services, Te Whatu Ora—Health New Zealand Waitematā, North Shore Hospital, Shakespeare Road, Takapuna, Auckland 0622, New Zealand

**Keywords:** heart rate variability (HRV), photoplethysmography (PPG), root mean square of successive R-R interval differences (RMSSDs), high-frequency HRV (HF-HRV), in-ear, fingertip, cardiac vagal tone

## Abstract

This paper presents a study undertaken to evaluate the sensor systems that were shortlisted to be used in the development of a portable respiratory-gated transcutaneous auricular vagus nerve stimulation (taVNS) system. To date, all published studies assessing respiratory-gated taVNS have been performed in controlled laboratory environments. This limitation arises from the reliance on non-portable sensing equipment, which poses significant logistical challenges. Therefore, we recognised a need to develop a portable sensor system for future research, enabling participants to perform respiratory-gated stimulation conveniently from their homes. This study aimed to measure the accuracy of an in-ear and a fingertip-based photoplethysmography (PPG) sensor in measuring cardiac vagal tone relevant heart rate variability (HRV) parameters of root mean square of successive R-R interval differences (RMSSDs) and the high-frequency (HF) component of HRV. Thirty healthy participants wore the prototype sensor equipment and the gold standard electrocardiogram (ECG) equipment to record beat-to-beat intervals simultaneously during 10 min of normal breathing and 10 min of deep slow breathing (DSB). Additionally, a stretch sensor was evaluated to measure its accuracy in detecting exhalation when compared to the gold standard sensor. We used Bland–Altman analysis to establish the agreement between the prototypes and the ECG system. Intraclass correlation coefficients (ICCs) were calculated to establish consistency between the prototypes and the ECG system. For the stretch sensor, the true positive rate (TPR), false positive rate (FPR), and false negative rate (FNR) were calculated. Results indicate that while ICC values were generally good to excellent, only the fingertip-based sensor had an acceptable level of agreement in measuring RMSSDs during both breathing phases. Only the fingertip-based sensor had an acceptable level of agreement during normal breathing in measuring HF-HRV. The study highlights that a high correlation between sensors does not necessarily translate into a high level of agreement. In the case of the stretch sensor, it had an acceptable level of accuracy with a mean TPR of 85% during normal breathing and 95% during DSB. The results show that the fingertip-based sensor and the stretch sensor had acceptable levels of accuracy for use in the development of the respiratory-gated taVNS system.

## 1. Introduction

Autonomic factors influencing changes in heart rate can be a result of the sympathetic nervous system (SNS) activity or the parasympathetic nervous system (PSNS) activity or their combination [1]. PSNS activity is modulated by the activity of the vagus nerve and is referred to as the cardiac vagal tone [1,2]. Heart rate variability (HRV) parameters that indicate rapid beat-to-beat changes and respiratory rhythms are recommended for indicating changes in the cardiac vagal tone [1,2]. There are various time domain and frequency domain HRV parameters of which the root mean square of successive R-R interval differences (RMSSDs) reflects vagal tone [3,4] and has a high correlation with the high-frequency (HF) component of HRV [3]. RMSSD is not influenced by respiration rate [5].

Activity in the vagal brainstem nuclei is cyclically modulated by respiration [6]. Recent functional magnetic resonance imaging (fMRI) studies [7,8] have shown exhalation-gated auricular vagal nerve stimulation evoked greater fMRI signal increase in brain regions associated with enhanced vagus nerve activity. Hence, we desired to build a taVNS system that synchronised the stimulation with the exhalation phase of the respiratory cycle and also measured changes in the cardiac vagal tone.

To measure the cardiac vagal tone, we needed to assess the accuracy of the photoplethysmography (PPG) heart rate sensors against a gold standard electrocardiogram (ECG) system [9]. Sensors using PPG technology were chosen as they are simple and inexpensive [10,11]. PPG technology uses non-invasive optical transceivers that are placed on the skin over the fingertip, wrist, forearm, or certain areas of the auricle, and infrared light is used to derive measures of heart rate and interbeat (R-R) intervals [10,11,12,13]. While PPG offers ease of use when compared to ECG systems, PPG measurements are more susceptible to motion artefacts [14,15,16,17,18] and peripheral circulation changes [14] than ECG. To counter these challenges, various publications recommend using appropriate signal processing methods and consideration of specific clinical applications [17,18,19,20]. The data processing section of this article discusses the custom algorithm developed for the chosen sensors to address the errors introduced by motion artefacts and intermittent fitment issues.

A detailed search of various PPG sensor components was carried out. Most feasible options included a fingertip-based or a wrist-based sensor. Camera-based PPG [21,22] options were not considered due to privacy concerns around the use of a camera, ambient lighting changes, and potential obstructions between the sensor and person that would make implementation difficult. Wrist-based sensors, commonly found in smartwatches, were ruled out due to the challenges with lower signal quality and higher susceptibility to noise when compared to fingertip-based sensors [23,24,25,26]. Therefore, fingertip and auricular (in-ear) HRV sensors were chosen.

To enable faster development of the prototype sensing system, off-the-shelf, well-established components were used for both sensors. The in-ear sensor option was preferred for the prototype system as the sensor and the stimulation electrodes could be housed in the same enclosure, making the system more compact. A literature review conducted for in-ear PPG sensor accuracy in measuring HRV during the development of this study protocol around mid-2022 yielded hundreds of results. Articles published in IEEE [27,28] discussed the development of a sensor system and algorithms for signal processing which required development beyond the scope of this study. A similar study by Tigges et al. [29] compared their custom in-ear sensor and a fingertip sensor with an ECG system and found a limited agreement for HRV parameters like HF HRV or low-frequency (LF)/HF ratio that quantify high frequent fluctuations in beat-to-beat intervals. They did not report on RMSSD. In summary, some publications [13,30] discussed in-ear PPG for measuring heart rate and even HRV [27,28,31]. However, the literature on the accuracy of measuring cardiac vagal tone-relevant HRV parameters using in-ear sensors was lacking. An in-ear sensor development kit from Valencell (Raleigh, NC, USA) [32] was used in this study.

Fingertip-based PPG sensors have been available for many years and are frequently used for measuring oxygen saturation levels [33,34], heart rate [35], and blood pressure [36]. Nonin Medical’s (Plymouth, MN, USA) [37] Xpod [38] system was used for the prototype system as it offers an easy-to-integrate interface for new or existing systems.

Various recent publications were reviewed [39,40,41,42,43] to shortlist potential sensor systems for respiration state detection. Of these, the depth camera and radar systems [42] offered a non-contact, non-wearable, and easy-to-use option. However, similar challenges with the use of camera-based PPG made them non-feasible for this study. A nasal airflow detection system [41] was ruled out due to the non-availability of commercial nasal airflow sensing kits. A stretch sensing system with a chest strap was considered the most feasible to implement. While there were commercial respiration state detection sensors like the Go-direct respiration belt (Vernier Science Education, Beaverton, OR, USA) [44], which was used as the gold standard in this study, they were locked by the use of their proprietary software and did not offer an option to integrate into our custom design. We used the stretch sensor kit from ElastiSense Sensor Technology (Aabenraa, Denmark) [45] as it supported integration into our design.

The primary goal of this study was to determine the accuracy of the prototype system’s PPG and respiration sensors when compared to the gold standard equipment. This goal was broken down into the following objectives:

1. To determine the accuracy of the in-ear and fingertip-based heart rate sensors in measuring HRV parameters that reflect changes in cardiac vagal tone;
2. To determine the accuracy of the stretch sensor in detecting the exhalation phase;
3. To determine whether a controlled breathing protocol is necessary to assist the respiration phase-detection method.

## 2. Materials and Methods

Ethical approval of the study was provided by the Auckland University of Technology Ethics Committee (AUTEC) on 12 October 2022 (reference number 22/275). All participants provided written informed consent.

### 2.1. Location

The study was conducted at the Health and Rehabilitation Research Institute on the Auckland University of Technology’s North Campus.

### 2.2. Recruitment Criteria

Participants were included if they were aged 18 years and over. Participants were excluded if they were unable to provide informed consent and/or comprehend fluent English, had a major neurological, psychological or cardiovascular disorder that could affect HRV or respiration, were currently taking any medications that could affect HRV or respiration, had an ear infection or piercings on the tragus, or were a current student of any of the study supervisors (according to ethics committee requirements).

### 2.3. Sample Size

A sample size of 30 participants was deemed sufficient for statistical analysis. The sample size was planned to support 80% statistical power for the intra-class correlation coefficient (ICC) for consistency between single measures from two instruments with a hypothesised ICC of 0.8 (good reliability) against a null value of 0.55 (poor reliability) at 5% type-I error rate [46].

### 2.4. Experimental Procedure

The participants were scheduled to attend a two-hour data collection session. Before the start of the session, they were briefed about the purpose of the study and the different sensors being tested.

**Figure 1:**
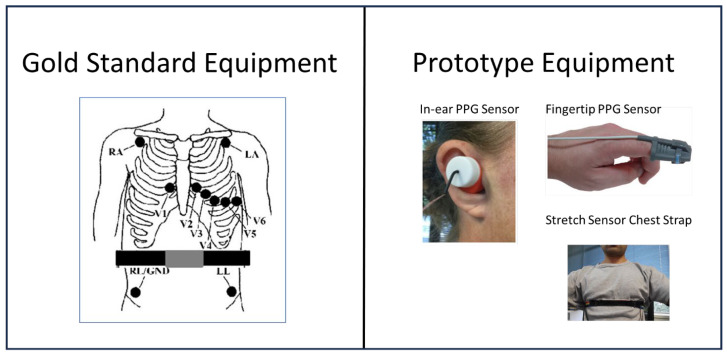
**Left**: Location of ECG electrodes and the gold standard respiration sensor strap. **Right**: Prototype equipment setup.

All data collection was conducted with the participants comfortably seated on a plinth in a temperature-controlled (21–22 °C) research laboratory. The participants were asked to remain still while their heart rate was monitored and captured continuously. Gold standard ECG measurements were obtained using the Custo Cardio 300 (Custo Med GmbH, Ottobrunn, Germany) 12 lead ECG system [47]. The electrode sites were prepared by cleaning the skin with an alcohol prep pad and red Dot 3M (3M New Zealand, Auckland, New Zealand) ECG electrodes [48] were placed as shown in Figure 1. ECG equipment was set up in default mode, with a sampling rate of 1000 Hz, to log R-R intervals. The participants wore the prototype system’s in-ear sensor in their right ear, such that the optical heart rate sensor was located on the inner concha. To ensure proper in-ear sensor fit, an appropriately sized earbud housing option (sizes supplied—small, medium, and large) was used. The fingertip-based sensor was worn on the tip of the index finger of the participant’s left hand as shown in Figure 1. A medium-sized finger-tip sleeve for finger thicknesses between 10 mm and 19 mm was used. The in-ear and fingertip-based sensors were set up to sample at their maximum rates of 250 Hz and 75 Hz, respectively. The in-ear sensor system did not allow logging the raw PPG waveform and only allowed logging the interbeat intervals while the fingertip-based sensor was set up to output the raw PPG waveform which was post-processed to calculate the interbeat intervals. The prototype sensor data were collected on the same computer system and were synchronised with the ECG’s computer system by a manual trigger which marked the start of 10 min windows. The trigger was actioned as soon as the ECG data logging started to keep the loss of synchronisation to a second which was considered a minor lag when comparing 600 s of datasets.

To measure the respiration state, a Go-direct respiration belt was worn over the participant’s abdomen just above the navel. The prototype stretch sensor was worn just above the Go-direct belt such that they were not overlapping and their sampling rates were set to 10 Hz.

### 2.5. Data Collection

Before beginning data collection, pilot testing was performed with 5 participants to calculate the average rest break duration between normal breathing and DSB phases as DSB has been shown to affect cardiac vagal tone. The results of pilot testing advised that 30 min was sufficient to wash out any changes to HRV.

**Figure 2:**
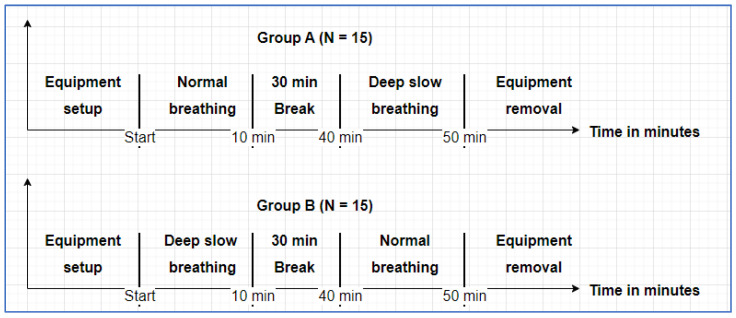
Data collection phase session timeline.

The participants were randomised using an online randomiser tool [49] into either Group A or Group B, wherein data were recorded for normal breathing or DSB first, respectively. The participants were seated on the plinth with the prototype sensors and gold standard devices attached. The participants were requested to stay as still as possible during data collection. The data were collected for 10 min during the normal breathing phase and the DSB phase. In between the two phases, there was a 30 min rest break during which no data were logged. Figure 2 shows the progression of the single data collection session for both groups.

### 2.6. Data Processing

Data from the ECG system and prototype (in-ear, fingertip-based) sensors were processed by a single script that extracted the R-R intervals (ECG data) and interbeat intervals (PPG data) from the specified time window and processed them through a series of checks and corrections. Various publications [50,51,52,53] were reviewed to understand different methods used for detecting outliers in the interval series data to complete the correction stage. Additionally, the in-ear dataset contained a signal quality score for each RR interval logged. The linear interpolation method was applied at the correction stage [54,55]. The following steps represent a customised algorithm that was developed and implemented:

1. Extract R-R/interbeat intervals for the 10 min window [56].
2. Stage 0: Apply for in-ear sensor dataset only.
3. Calculate the average signal quality score and mark the dataset to be excluded from statistical analysis if the average score is less than 70 out of 100 to avoid noisy sensor data from affecting the overall accuracy calculations.
  - Identify each R-R interval with a signal quality score of less than 40 and mark it for interpolation.
  - Interpolate each identified interval using the linear interpolation technique.
4. Stage 1: Identify beat-per-minute (BPM) outliers with intervals less than 400 milliseconds (150 BPM) or more than 1700 milliseconds (35 BPM).
5. Interpolate each identified interval using the linear interpolation technique.
6. Stage 2: Identify more outliers by checking whether any of the following additional checks are met:
  - Is the current interval equal to the previous interval?
  - Does the current interval differ by more than 20% of the mean of the previous and the next interval [57]? If either the previous interval and/or the next interval is outside the 3 standard deviations, use the mean value of the R-R series instead.
  - Is the current interval outside of 3 standard deviations [53,58] of the intervals in the current dataset?
7. Interpolate any intervals identified in Stage 2 checks using the linear interpolation technique.
8. Calculate HRV parameters.

**Figure 3:**
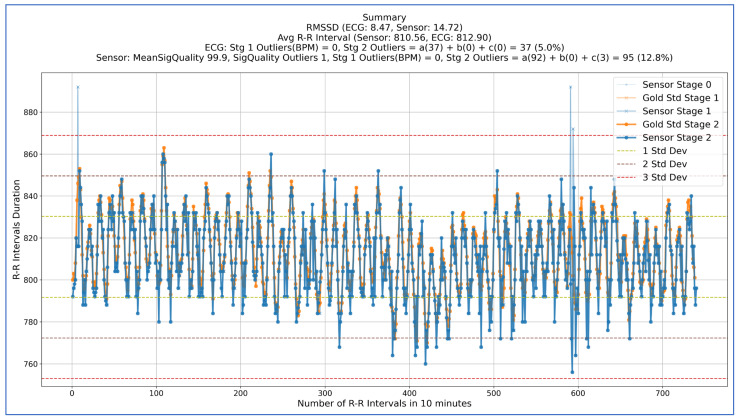
Visual representation of the R-R interval outlier detection and correction algorithm. Dashed lines represent 1, 2, and 3 standard deviations of the in-ear sensor dataset. Blue lines represent in-ear data and lighter shaded lines represent data before correction. Orange lines represent ECG data. Stage 0 lines represent the intervals before any correction. Stage 1 lines represent the intervals adjusted after filtering BPM outliers. Stage 2 lines represent the intervals adjusted after filtering outliers that met the criteria listed in Step 6 of the algorithm.

As an example, Figure 3 shows a visualisation of the above algorithm when comparing one participant’s ECG dataset with the corresponding in-ear data during the deep slow breathing phase. The horizontal dashed lines represent 1 (green), 2 (brown), and 3 (red) standard deviations. There were no BPM outliers identified in either dataset. In this example, the mean signal quality for the in-ear sensor was 99.9% with only one signal quality outlier. At Stage 2 of the checks, there were 37 beats (5.0%) identified in the ECG dataset and 95 beats (12.8%) identified in the in-ear dataset as outliers. The average R-R intervals calculated were 812 ms and 810.5 ms for ECG and in-ear, respectively. However, the RMSSD values were 8.47 ms and 14.72 ms, respectively, suggesting that even if the data are time synchronised and look very similar, the error margin in RMSSD calculations can be notable for low-range RMSSD values.

Once the data were cleaned, an open-source Python package hrv-analysis (version 1.0.4) [59] was used for calculating RMSSD and HF-HRV parameters (Step 8). In the case of the in-ear sensor, data were excluded from 3 participants for the DSB phase and 2 participants from the normal breathing phase due to their mean signal quality score being less than 70%. No data were excluded from the normal breathing phase. In the case of the fingertip-based sensor, data were excluded from one participant for the normal breathing phase due to the total R-R interval count being out by more than 10% when compared to the corresponding ECG dataset. This left complete data from 27 participants (DSB, in-ear sensor, signal quality scores averaging 91.6 ± 10.2 with scores ranging from 70.1 to 100), 28 participants (normal breathing, in-ear sensor, signal quality scores averaging 96.1 ± 5.2 with score ranging from 80 to 100), 30 participants (DSB, finger sensor, intervals count averaging 99.5% ± 1.1 with scores ranging from 93.7 to 100), and 29 participants (normal breathing, finger sensor, intervals count averaging 99.8% ± 0.2 with scores ranging from 98.7 to 100), respectively (Supplementary: Data S4).

### 2.7. Statistical Analysis

#### 2.7.1. HRV Parameters

Bland–Altman analysis was conducted to evaluate the accuracy of the HRV parameters obtained from the in-ear and fingertip-based sensor against the same parameters obtained from the ECG system [60] (Supplementary: Data S1). Limits of agreement (LOAs) between the two instruments were reported from the data of the non-excluded participants in each dataset. The LOAs were expressed as a percentage of the mean parameter value (LOA%) and interpreted as excellent (<5%), good (5–9.9%), and acceptable (10–30%) [61,62]. The following steps represent the method used to calculate the LOAs (Supplementary: Data S2). The HRV parameter of RMSSD is mentioned; however, the same steps were applied to HF-HRV calculations as well.

1. N data points, 1 per participant, were extracted for ECG and the prototype sensor. Each data point represented the corresponding RMSSD for the respective 10 min breathing protocols.
2. Mean RMSSD values of the ECG and prototype sensor for each corresponding data point were calculated.
3. The difference values between the ECG and prototype sensor for each corresponding data point were calculated.
4. The bias was calculated as a mean of all the difference values in Step 3.
5. The upper and lower bounds of the LOA were calculated as bias ± 1.96 x standard deviation of differences in Step 3.

The intra-class correlation coefficient (ICC) was also computed from the variance components as a relative measure of consistency between single measures from the two instruments. ICC was interpreted as excellent (0.90–1), good (0.750–0.899), moderate (0.50–0.749), and poor (0–0.499) [63]. Based on the guidelines for selecting and reporting ICC for reliability research [64,65], a two-way mixed-effects model was chosen to report interrater reliability. The two-way mixed-effects model was used to represent reliability for specific raters rather than for generalising to other raters with similar characteristics [64,65]. Interrater reliability reflects the variation between two or more raters measuring the same subjects. Further, single rater ICC type was chosen as the measurements being compared were not mean values of multiple raters; rather, they were single measures (10 min readings) taken by two different raters (ECG and each prototype sensor). In the case of definition, Trevathan [65] argued that if the purpose is to measure how identical the ratings are for each subject, an absolute agreement definition should be favoured. Also, ICCs calculated by absolute agreement are typically smaller than the consistency definition. Hence, measures for both definitions are shared to present a comprehensive comparison of the devices. Consistency definition has implications for applications where a correlation with the gold standard is sufficient. In contrast, the absolute agreement definition is useful for applications which require a minimal difference in scores compared to the gold standard. For this study, ICC definitions of consistency were used and the absolute agreement was evaluated by Bland–Altman analysis.

#### 2.7.2. Respiration Phase Detection

Respiration phase detection accuracy was calculated by comparing the Go-direct respiration belt waveform data with the prototype stretch sensor data. The datasets were compared after the session start timestamps were synchronised. An acceptable exhalation phase match window of 1.5 s for normal breathing and 2 s for DSB was deemed long enough for a true match given our intended application. The ground-truth windows were obtained from the Go-direct belt waveform. A custom exhalation phase detection algorithm marked the start and the end of the exhalation window for each respiration cycle in the gold standard and prototype datasets. If the stretch sensor dataset’s exhalation windows overlapped the corresponding ground-truth dataset’s exhalation windows for a minimum of 1.5 s during normal breathing or a minimum of 2 s during DSB, they were marked as a match. Otherwise, the window was counted as a miss. Exhalation windows that were less than the minimum length of 1.5 s (normal breathing) or 2 s (DSB) in the gold standard dataset were excluded from the calculations.

For each participant dataset:

1. The true positive rate (TPR) was obtained by calculating the percentage of exhalation windows in the stretch sensor dataset that matched the corresponding valid ground-truth exhalation windows;
2. The false positive rate (FPR) was reported by calculating the percentage of exhalation windows in the stretch sensor data that were additional and did not align with the corresponding valid ground-truth exhalation windows;
3. The false negative rate (FNR) was reported by calculating the percentage of missing peaks in the stretch sensor data out of the corresponding total valid ground-truth peaks.

Mean and 95% confidence intervals (CIs) [66] for these rates were also calculated. While there is no established reference for acceptable TPR, we interpreted a mean TPR > 80% and FPR < 20%, inferred with 95% CIs, as an acceptable level of accuracy for the prototype sensor and our intended application.

## 3. Results

### 3.1. Participants’ Characteristics

Thirty participants with a mean age of 37 years (range 18–71; 18 female) were recruited. Most of the participants were European (16); however, there were also Asian (9), Māori (3), and Pasifika (2) participants.

### 3.2. HRV Parameters

Table 1 shows the results of the Bland–Altman analysis. HF-HRV values are represented in normalised units (HFNU) as normalisation minimises the impact of changes in total power on the values of low-frequency and HF components [67,68]. The LOA error percentage values that were within the acceptable range (≤30%) are highlighted in green colour. In the case of HFNU, none of the other error percentages for measuring HFNU, except for the fingertip-based sensor’s borderline value of 29.70% during normal breathing, were within the acceptable range. The in-ear sensor’s lowest error percentage of 33.49%, when evaluating the DSB dataset, was still outside the acceptable range.

**Figure 4:**
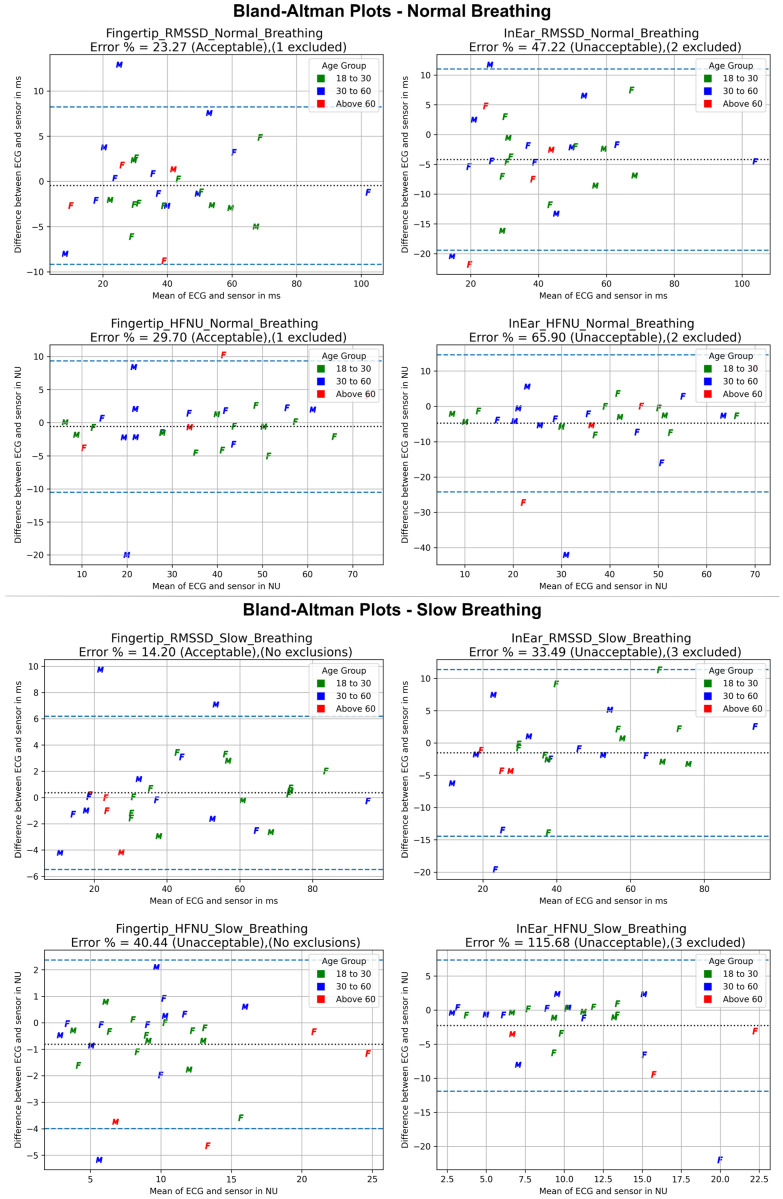
Bland–Altman plots for the fingertip-based and the in-ear sensors are shown on the left and right, respectively. The titles associated with the subplots mention the HRV parameter, breathing protocol, error percentage (LOA), and exclusions. In each subplot, each point, representing each participant, is marked as “M” for male and “F” for female to denote gender. Each data point represents the mean of the measurements (ECG and the respective sensor) for each participant. The colours represent the age bracket of the participant with green denoting age bracket 18–30, blue denoting 30–60, and red denoting above 60. The black dotted line in the middle represents the mean difference or the bias and the dashed blue lines represent the lower and upper bounds of LOAs.

Figure 4 shows the Bland–Altman plots for each sensor’s data during normal breathing in the top half and during DSB in the bottom half.

For ICC calculations for the HRV data, the ECG and prototype sensors were the raters. HRV parameters of RMSSDs and HFNUs were the ratings, and the subjects were the participants. Statistical analysis was performed using the Pingouin package (version 0.5.3) [69] in Python and the results were cross-checked with the output from IBM SPSS Statistics for Windows (version 29) [70] (Supplementary: Data S5, Data S6). As mentioned in the Methods section, results for the definitions of consistency and absolute agreement (Supplementary: PDF S1, PDF S2) for the two-way mixed-effects model and the single rater type are shown in Table 2 and Table 3, respectively. As per the ICC guidelines [64], based on the 95% confidence intervals, the in-ear coefficients for measuring HFNUs are indicative of good reliability (between 0.75 and 0.90) during normal breathing and moderate reliability (between 0.50 and 0.75) during DSB. The rest of the values indicate excellent reliability (>0.90).

### 3.3. Respiration Phase Measurement

Table 4 shows the TPR, FPR, and FNR values for each breathing phase for all 30 participants (Supplementary: Data S3). The mean inferred rates were taken as the lower bound of the 95% CIs for TPR and the upper bound for FPR.

## 4. Discussion

The primary goal of this study was to evaluate the accuracy of the prototype sensors against gold standard equipment. The results suggest that the prototype sensors used in conjunction with a custom data processing algorithm, except the in-ear sensor, can provide an acceptable level of accuracy.

In terms of ICC values, both prototype sensors showed excellent correlation when measuring RMSSD. This suggests that there is excellent consistency between the two prototype sensors and the ECG system. However, Bland and Altman have criticised the use of ICC when assessing agreement between two instruments [71]. If the variance between subjects is high, which was the case in the range of HRV values of the participants, ICC results are biased towards higher values [72]. Hence, correlation coefficients denote high reliability but not necessarily high agreement in this case.

Only the fingertip-based sensor measured RMSSD with an acceptable level of agreement (LOA error % less than 30%) [61,62] during both breathing phases. Based on this outcome, the fingertip-based sensor can be used with either breathing protocol.

HFNU measurements had a high percentage of error during DSB as the protocol used six breaths/minute (0.1 Hz) cues, which was outside the HF band range (0.15 to 0.4 Hz). During normal breathing, the error percentage for measuring HFNUs using the fingertip-based sensor was just within the acceptable range (29.70%), whereas the in-ear sensor’s 65.90% was well above the acceptable limit range. Hence, only the fingertip-based sensor was sufficiently accurate in measuring HFNUs during the normal breathing protocol.

Overall, the fingertip-based sensor showed lower LOA error % and higher ICC values compared to the in-ear sensor and had an acceptable agreement when compared to the gold standard ECG system. The 75 Hz sampling rate is a limiting factor for the fingertip-based sensor’s accuracy as it provides a 13.3 ms resolution. This causes a loss of precision when measuring RMSSD for recording values less than 20 ms. Various publications suggest that RMSSD reduces with age and normative values can be below 20 ms, even in healthy populations above the age of 50 years [73,74,75]. However, the sensor showed excellent correlation when compared to the gold standard, suggesting that trends in changes in RMSSD values can be captured even with a higher error percentage at lower RMSSD values.

In the case of respiration state detection, the stretch sensor had an acceptable level of accuracy during both breathing protocols. The mean TPR with 95% CI (85%) during normal breathing would ensure that the stimulation would be imparted during at least 85% of the exhalations. While it had a better mean TPR (95%) during DSB, the use of the DSB cue was only a backup option in case the strap failed to detect the start of exhalation with sufficient accuracy. Whether these levels of TPR/FPR are efficacious or not is an open question that can only be resolved with an experimental study of respiratory-gated taVNS.

### Strengths and Limitations

This is one of the few studies comparing the accuracy of data reported by an in-ear and fingertip-based sensor for calculating HRV parameters (RMSSD and HFNUs) when compared to a gold standard 12-lead ECG system. The fingertip-based sensor used in this study is a clinical device for measuring pulse oximetry and heart rate only [38]. We utilised additional data from the sensor to derive RR intervals and expanded its applicability. A custom signal-cleaning algorithm, like the one developed during this study, is essential in improving the accuracy of HRV estimates.

A few limitations of this current study should be noted. The sample size was relatively small (30), although the participants belonged to varied age groups and ethnicities. A larger sample size is required to further analyse whether there are any differences in sensor accuracy based on gender, age, and ethnicity. Based on this study’s observations, the low sampling rate for the fingertip-based sensor would result in a higher error percentage for participants with RMSSD values lower than 20 ms. Future studies may look at the impact of low sampling rate PPG systems in measuring HRV in people with low RMSSD. The accuracy of the sensors used is likely dependent on the proper fitting and minimal movement of the hand/finger (for the fingertip-based sensor) or the head/jaw (for the in-ear sensor). Finally, the estimates here only apply to average values over a 10 min window, as this suited our intended application of taVNS. The consistency and agreement between sensors remains unknown over shorter periods and may not be the same.

## 5. Conclusions

This study set out to determine the accuracy of the prototype in-ear and fingertip-based sensors in measuring HRV parameters that reflect changes in cardiac vagal tone. The results show that the fingertip-based sensor is the most accurate in measuring RMSSD during either breathing protocol. Next, it set out to determine the accuracy of the respiration stretch sensor in detecting the exhalation phase. The results show that the respiration stretch sensor had an acceptable level of accuracy during either breathing protocol. Finally, we wanted to determine whether the DSB protocol was necessary to assist with respiration phase detection, and the results show that the strap has an acceptable level of accuracy during normal breathing; hence, the DSB protocol is not necessary.

## Figures and Tables

**Table 1:** Bland–Altman analysis summary for the in-ear and fingertip-based sensor data when compared to the ECG data in measuring RMSSD in milliseconds and HF-HRV in normalised units (HFNUs). Values in the acceptable range are highlighted in bold font.

*N*	Sensor	Breathing	Parameter	Mean Difference	LOA Upper Bound	LOA Lower Bound	LOA %
27	In-Ear	DSB	RMSSD	−1.53	11.40	−14.46	33.49
27	In-Ear	DSB	HFNU	−2.29	7.34	−11.93	115.68
28	In-Ear	Normal	RMSSD	−4.20	11.02	−19.42	47.22
28	In-Ear	Normal	HFNU	−4.84	14.57	−24.26	65.90
30	Finger	DSB	RMSSD	0.36	6.19	−5.47	**14.20**
30	Finger	DSB	HFNU	−0.81	2.37	−3.99	40.44
29	Finger	Normal	RMSSD	−0.47	8.23	−9.17	**23.27**
29	Finger	Normal	HFNU	−0.60	9.34	−10.54	**29.70**

**Table 2:** Intraclass correlation coefficients, **for consistency**, for the in-ear and fingertip-based sensor data when compared to the ECG data in measuring RMSSDs in milliseconds and HF-HRV in normalised units (HFNUs).

*N*	Sensor	Breathing	Parameter	ICC	*p*-Value	Confidence Intervals
27	In-Ear	DSB	RMSSD	0.95	<0.001	[0.90 0.98]
27	In-Ear	DSB	HFNU	0.58	<0.001	[0.20 0.76]
28	In-Ear	Normal	RMSSD	0.92	<0.001	[0.76 0.96]
28	In-Ear	Normal	HFNU	0.84	<0.001	[0.61 0.92]
30	Finger	DSB	RMSSD	0.99	<0.001	[0.98 1.00]
30	Finger	DSB	HFNU	0.95	<0.001	[0.85 0.97]
29	Finger	Normal	RMSSD	0.98	<0.001	[0.95 0.99]
29	Finger	Normal	HFNU	0.96	<0.001	[0.92 0.98]

**Table 3:** Intraclass correlation coefficients, **for absolute agreement**, for the in-ear and fingertip-based sensor data when compared to the ECG data in measuring RMSSDs in milliseconds and HF-HRV in normalised units (HFNUs).

*N*	Sensor	Breathing	Parameter	ICC	*p*-Value	Confidence Intervals
27	In-Ear	DSB	RMSSD	0.95	<0.001	[0.90 0.98]
27	In-Ear	DSB	HFNU	0.54	<0.001	[0.24 0.78]
28	In-Ear	Normal	RMSSD	0.91	<0.001	[0.83 0.97]
28	In-Ear	Normal	HFNU	0.82	<0.001	[0.67 0.91]
30	Finger	DSB	RMSSD	0.99	<0.001	[0.98 1.00]
30	Finger	DSB	HFNU	0.94	<0.001	[0.83 0.97]
29	Finger	Normal	RMSSD	0.98	<0.001	[0.95 0.99]
29	Finger	Normal	HFNU	0.96	<0.001	[0.91 0.98]

**Table 4:** Stretch sensor’s accuracy data when compared to the ground-truth data.

Breathing	Parameter	Mean	Lower Bound	Upper Bound	Mean Inferred with 95% CI
Normal	TPR	90	85	94	85
Normal	FPR	1	0	1	1
Normal	FNR	10	5	14	14
DSB	TPR	97	94	98	95
DSB	FPR	3	1	4	4
DSB	FNR	3	1	5	5

## Data Availability

The data presented in this study are available from the corresponding author upon request.

## Supplementary Materials

The following supporting information can be downloaded at: https://www.mdpi.com/article/10.3390/s25051485/s1. PDF S1: SPSS ICC results for fingertip sensor; PDF S2: SPSS ICC results for ear sensor; Data S1: HRV values for each participant from each device; Data S2: Mean of ECG and each sensor’s HRV values; Data S3: Respiration data summary; Data S4: List of exclusions from respective datasets; Data S5: Data formatted for processing in SPSS for fingertip sensor; Data S6: Data formatted for processing in SPSS for in-ear sensor.

## Abbreviations

The following abbreviations are used in this manuscript:

PPG	Photoplethysmography
HRV	Heart Rate Variability
RMSSD	Root Mean Square of Successive R-R Interval Differences
HF	High Frequency
HFNU	High-Frequency Normalised Units
ECG	Electrocardiogram
DSB	Deep Slow Breathing
ICC	Intraclass Correlation Coefficients
TPR	True Positive Rate
FPR	False Positive Rate
FNR	False Negative Rate
taVNS	Transcutaneous Auricular Vagus Nerve Stimulation
CIs	Confidence Intervals
LOA	Limits of Agreement

## References

[1] Task Force of the European Society of Cardiology the North American Society of Pacing Electrophysiology (1996). Heart Rate Variability. Circulation.

[2] Chapleau M.W., Sabharwal R. (2010). Methods of assessing vagus nerve activity and reflexes. Heart Fail. Rev..

[3] Kleiger R.E., Stein P.K., Bigger J.T. (2005). Heart rate variability: Measurement and clinical utility. Ann. Noninvasive Electrocardiol..

[4] Thayer J.F., Lane R.D. (2000). A model of neurovisceral integration in emotion regulation and dysregulation. J. Affect. Disord..

[5] Hill L.K., Siebenbrock A. (2009). Are all measures created equal? Heart rate variability and respiration-biomed 2009. Biomed. Sci. Instrum..

[6] Napadow V., Edwards R.R., Cahalan C.M., Mensing G., Greenbaum S., Valovska A., Li A., Kim J., Maeda Y., Park K. (2013). Evoked Pain Analgesia in Chronic Pelvic Pain Patients using Respiratory-gated Auricular Vagal Afferent Nerve Stimulation. Pain Med..

[7] Garcia R.G., Lin R.L., Lee J., Kim J., Barbieri R., Sclocco R., Wasan A.D., Edwards R.R., Rosen B.R., Hadjikhani N. (2017). Modulation of brainstem activity and connectivity by respiratory-gated auricular vagal afferent nerve stimulation in migraine patients. Pain.

[8] Sclocco R., Garcia R.G., Kettner N.W., Isenburg K., Fisher H.P., Hubbard C.S., Ay I., Polimeni J.R., Goldstein J., Makris N. (2019). The influence of respiration on brainstem and cardiovagal response to auricular vagus nerve stimulation: A multimodal ultrahigh-field (7T) fMRI study. Brain Stimul..

[9] Shaffer F., Ginsberg J.P. (2017). An Overview of Heart Rate Variability Metrics and Norms. Front. Public Health.

[10] Saquib N., Papon M.T.I., Ahmad I., Rahman A. Measurement of heart rate using photoplethysmography. Proceedings of the 2015 International Conference on Networking Systems and Security (NSysS).

[11] Blok S., Piek M.A., Tulevski I.I., Somsen G.A., Winter M.M. (2021). The accuracy of heartbeat detection using photoplethysmography technology in cardiac patients. J. Electrocardiol..

[12] Vescio B., Salsone M., Gambardella A., Quattrone A. (2018). Comparison between Electrocardiographic and Earlobe Pulse Photoplethysmographic Detection for Evaluating Heart Rate Variability in Healthy Subjects in Short- and Long-Term Recordings. Sensors.

[13] Passler S., Müller N., Senner V. (2019). In-Ear Pulse Rate Measurement: A Valid Alternative to Heart Rate Derived from Electrocardiography?. Sensors.

[14] Gonçalves H., Pinto P., Silva M., Ayres-de-Campos D., Bernardes J. (2016). Electrocardiography versus photoplethysmography in assessment of maternal heart rate variability during labor. SpringerPlus.

[15] Bolanos M., Nazeran H., Haltiwanger E. Comparison of heart rate variability signal features derived from electrocardiography and photoplethysmography in healthy individuals. Proceedings of the 2006 International Conference of the IEEE Engineering in Medicine and Biology Society.

[16] Lu G., Yang F., Taylor J.A., Stein J.F. (2009). A comparison of photoplethysmography and ECG recording to analyse heart rate variability in healthy subjects. J. Med. Eng. Technol..

[17] Georgieva-Tsaneva G., Georgieva-Tsaneva G.N., Gospodinova E. (2021). Comparative Heart Rate Variability Analysis of ECG, Holter and PPG Signals. Int. J. Adv. Comput. Sci. Appl..

[18] Rossi A., Pedreschi D., Clifton D.A., Morelli D. (2020). Error Estimation of Ultra-Short Heart Rate Variability Parameters: Effect of Missing Data Caused by Motion Artifacts. Sensors.

[19] Mejia-Mejia E., May J.M., Kyriacou P.A. Effect of Filtering of Photoplethysmography Signals in Pulse Rate Variability Analysis. Proceedings of the 2021 43rd Annual International Conference of the IEEE Engineering in Medicine & Biology Society (EMBC).

[20] Esgalhado F., Batista A., Vassilenko V., Russo S., Ortigueira M. (2022). Peak Detection and HRV Feature Evaluation on ECG and PPG Signals. Symmetry.

[21] Trumpp A., Lohr J., Wedekind D., Schmidt M., Burghardt M., Heller A.R., Malberg H., Zaunseder S. (2018). Camera-based photoplethysmography in an intraoperative setting. Biomed. Eng. Online.

[22] Raposo A., Da Silva H.P., Sanches J. Camera-based Photoplethysmography (cbPPG) using smartphone rear and frontal cameras: An experimental study. Proceedings of the 2021 43rd Annual International Conference of the IEEE Engineering in Medicine & Biology Society (EMBC).

[23] Rajala S., Lindholm H., Taipalus T. (2018). Comparison of photoplethysmogram measured from wrist and finger and the effect of measurement location on pulse arrival time. Physiol. Meas..

[24] Pradhan N., Rajan S., Adler A. (2019). Evaluation of the signal quality of wrist-based photoplethysmography. Physiol. Meas..

[25] Castaneda D., Esparza A., Ghamari M., Soltanpur C., Nazeran H. (2018). A review on wearable photoplethysmography sensors and their potential future applications in health care. Int. J. Biosens. Bioelectron..

[26] Almarshad M.A., Islam M.S., Al-Ahmadi S., Bahammam A.S. (2022). Diagnostic Features and Potential Applications of PPG Signal in Healthcare: A Systematic Review. Healthcare.

[27] Lueken M., Feng X., Venema B., Misgeld B.J.E., Leonhardt S. Photoplethysmography-based in-ear sensor system for identification of increased stress arousal in everyday life. Proceedings of the 2017 IEEE 14th International Conference on Wearable and Implantable Body Sensor Networks (BSN).

[28] Pedrana A., Comotti D., Re V., Traversi G. (2020). Development of a Wearable In-Ear PPG System for Continuous Monitoring. IEEE Sens. J..

[29] Tigges T., Büchler T., Pielmuş A., Klum M., Feldheiser A., Hunsicker O., Orglmeister R. (2018). Assessment of in-ear photoplethysmography as a surrogate for electrocardiography in heart rate variability analysis. World Congress on Medical Physics and Biomedical Engineering 2018: 3–8 June 2018, Prague, Czech Republic (Vol. 2).

[30] Winokur E.S., Da He D., Sodini C.G. A Wearable Vital Signs Monitor at the Ear for Continuous Heart Rate and Pulse Transit Time Measurements. Proceedings of the 2012 Annual International Conference of the IEEE Engineering in Medicine and Biology Society.

[31] Ferlini A., Montanari A., Min C., Li H., Sassi U., Kawsar F. (2021). In-Ear PPG for Vital Signs. IEEE Pervasive Comput..

[32] Benchmark-Valencell. https://valencell.com/benchmark/.

[33] Elgendi M. (2012). On the Analysis of Fingertip Photoplethysmogram Signals. Curr. Cardiol. Rev..

[34] Tamura T. (2019). Current progress of photoplethysmography and SPO2 for health monitoring. Biomed. Eng. Lett..

[35] Aarthi Y., Karthikeyan B., Raj N.P., Ganesan M. Fingertip Based Estimation of Heart Rate Using Photoplethysmography. Proceedings of the 2019 5th International Conference on Advanced Computing & Communication Systems (ICACCS).

[36] Paliakaite B., Charlton P.H., Rapalis A., Plusciauskaite V., Piartli P., Kaniusas E., Marozas V. Blood Pressure Estimation Based on Photoplethysmography: Finger Versus Wrist. Proceedings of the 2021 Computing in Cardiology (CinC).

[37] About-Nonin. https://www.nonin.com/about/.

[38] Xpod^®^ 3012LP-Nonin. https://www.nonin.com/products/xpod/.

[39] Roudjane M., Khalil M., Miled A., Messaddeq Y. (2018). New Generation Wearable Antenna Based on Multimaterial Fiber for Wireless Communication and Real-Time Breath Detection. Photonics.

[40] Chu M., Nguyen T., Pandey V., Zhou Y., Pham H.N., Bar-Yoseph R., Radom-Aizik S., Jain R., Cooper D.M., Khine M. (2019). Respiration rate and volume measurements using wearable strain sensors. npj Digit. Med..

[41] da Costa T.D., de Fatima Fernandes Vara M., Cristino C.S., Zanella T.Z., Neto G.N.N., Nohama P. (2019). Breathing Monitoring and Pattern Recognition with Wearable Sensors. Wearable Devices-the Big Wave of Innovation.

[42] He S., Han Z., Iglesias C., Mehta V., Bolic M. (2022). A Real-Time Respiration Monitoring and Classification System Using a Depth Camera and Radars. Front. Physiol..

[43] Mansy H.A., Azad M.K., Gamage P.T., Sandler R.H. (2018). Detection of respiratory phase and rate from chest surface measurements. J. Appl. Biotechnol. Bioeng..

[44] Go Direct^®^ Respiration Belt-Vernier. https://www.vernier.com/product/go-direct-respiration-belt/.

[45] Stretch Sensor Kit|ElastiSense Sensor Technology. https://elastisense.com/product/stretch-sensor-kit/.

[46] Shoukri M.M., Asyali M.H., Donner A. (2004). Sample size requirements for the design of reliability study: Review and new results. Stat. Methods Med. Res..

[47] Custo-Med Cardio 300 ECG-Promed|Medical Equipment and Supplies for Ireland. https://www.promed.ie/custo-cardio-300bt-ecg-for-new-custo-med-users-kit.html.

[48] 3M™ Red Dot™ ECG Monitoring Electrodes, Foam, Diaphoretic, 2560, 50 Dots/Bag, 20 Bags/Case|3M New Zealand. https://www.3mnz.co.nz/3M/en_NZ/p/d/v000154410/.

[49] Randomly Assign Subjects to Treatment Groups. https://www.graphpad.com/quickcalcs/randomize1/.

[50] Mejia-Mejia E., Kyriacou P.A. Outlier Management for Pulse Rate Variability Analysis from Photoplethysmographic Signals. Proceedings of the 2022 44th Annual International Conference of the IEEE Engineering in Medicine & Biology Society (EMBC).

[51] Plews D.J., Scott B., Altini M., Wood M., Kilding A.E., Laursen P.B. (2017). Comparison of heart-rate-variability recording with smartphone photoplethysmography, polar H7 chest strap, and electrocardiography. Int. J. Sports Physiol. Perform..

[52] Deegan B.M.T., O’Connor M., Lyons D., ÓLaighin G. A new blood pressure and heart rate signal analysis technique to assess Orthostatic Hypotension and its subtypes. Proceedings of the 2007 29th Annual International Conference of the IEEE Engineering in Medicine and Biology Society.

[53] Kemper K.J., Hamilton C., Atkinson M. (2007). Heart Rate Variability: Impact of Differences in Outlier Identification and Management Strategies on Common Measures in Three Clinical Populations. Pediatr. Res..

[54] Peltola M.A. (2012). Role of editing of R-R intervals in the analysis of heart rate variability. Front. Physiol..

[55] Morelli D., Rossi A., Cairo M., Clifton D.A. (2019). Analysis of the Impact of Interpolation Methods of Missing RR-intervals Caused by Motion Artifacts on HRV Features Estimations. Sensors.

[56] Bourdillon N., Schmitt L., Yazdani S., Vesin J.M., Millet G.P. (2017). Minimal window duration for accurate HRV recording in athletes. Front. Neurosci..

[57] Karlsson M., Hörnsten R., Rydberg A., Wiklund U. (2012). Automatic filtering of outliers in RR intervals before analysis of heart rate variability in Holter recordings: A comparison with carefully edited data. Biomed. Eng. Online.

[58] Pukelsheim F. (1994). The three sigma rule. Am. Stat..

[59] Champseix R., Ribiere L., Le Couedic C. (2021). A Python Package for Heart Rate Variability Analysis and Signal Preprocessing. J. Open Res. Softw..

[60] Bland J.M., Altman D.G. (1999). Measuring agreement in method comparison studies. Stat. Methods Med. Res..

[61] Odor P.M., Bampoe S., Cecconi M. (2017). Cardiac Output Monitoring: Validation Studies–how Results Should be Presented. Curr. Anesthesiol. Rep..

[62] Cecconi M., Rhodes A., Poloniecki J., Della Rocca G., Grounds R.M. (2009). Bench-to-bedside review: The importance of the precision of the reference technique in method comparison studies–with specific reference to the measurement of cardiac output. Crit. Care.

[63] Portney L.G., Watkins M.P. (2008). Foundations of Clinical Research: Applications to Practice.

[64] Koo T.K., Li M.Y. (2016). A Guideline of Selecting and Reporting Intraclass Correlation Coefficients for Reliability Research. J. Chiropr. Med..

[65] Trevethan R. (2017). Intraclass correlation coefficients: Clearing the air, extending some cautions, and making some requests. Health Serv. Outcomes Res. Methodol..

[66] Cumming G. (2009). Inference by eye: Reading the overlap of independent confidence intervals. Stat. Med..

[67] Burr R.L. (2007). Interpretation of Normalized Spectral Heart Rate Variability Indices In Sleep Research: A Critical Review. Sleep.

[68] Malik M., Bigger J.T., Camm A.J., Kleiger R.E., Malliani A., Moss A.J., Schwartz P.J. (1996). Heart rate variability. Standards of measurement, physiological interpretation, and clinical use. Eur. Heart J..

[69] Vallat R. (2018). Pingouin: Statistics in Python. J. Open Source Softw..

[70] IBM Corp (2023). IBM SPSS Statistics for Windows.

[71] Zaki R., Bulgiba A., Ismail R., Ismail N.A. (2012). Statistical Methods Used to Test for Agreement of Medical Instruments Measuring Continuous Variables in Method Comparison Studies: A Systematic Review. PLoS ONE.

[72] Bruton A., Conway J.H., Holgate S.T. (2000). Reliability: What is it, and how is it measured?. Physiotherapy.

[73] Voss A., Schroeder R., Heitmann A., Peters A., Perz S. (2015). Short-Term Heart Rate Variability—Influence of Gender and Age in Healthy Subjects. PLoS ONE.

[74] Tegegne B.S., Man T., van Roon A.M., Snieder H., Riese H. (2020). Reference values of heart rate variability from 10-second resting electrocardiograms: The Lifelines Cohort Study. Eur. J. Prev. Cardiol..

[75] Nunan D., Sandercock G.R.H., Brodie D.A. (2010). A Quantitative Systematic Review of Normal Values for Short-Term Heart Rate Variability in Healthy Adults. Pacing Clin. Electrophysiol..

