# Supplementary material for: Evaluation of In-Ear and Fingertip-Based Photoplethysmography Sensors for Measuring Cardiac Vagal Tone Relevant Heart Rate Variability Parameters
Source: Sensors (Basel). 2025 Feb 28;25(5):1485. doi: 10.3390/s25051485 (PMC11902391; doi:10.3390/s25051485)
Supplement: Supplementary file 1 [file sensors-25-01485-s001.zip › PDFS1.pdf]

DSB_RMSSD_CONS

Scale: ALL VARIABLES

Case Processing Summary

		N	%
Cases	Valid	30	100.0
	Excluded[a]	0	.0
	Total	30	100.0

a. Listwise deletion based on all variables in the procedure.

Reliability Statistics

Cronbach's Alpha	N of Items
.996	2

Intraclass Correlation Coefficient

	Intraclass Correlation[b]	95% Confidence Interval		F Test with True Value 0		
		Lower Bound	Upper Bound	Value	df1	df2
Single Measures	.992[a]	.982	.996	234.813	29	29
Average Measures	.996[c]	.991	.998	234.813	29	29

Intraclass Correlation Coefficient

	F Test with ..
	Sig
Single Measures	<.001
Average Measures	<.001

Two-way mixed effects model where people effects are random and measures effects are fixed.

a. The estimator is the same, whether the interaction effect is present or not.

b. Type C intraclass correlation coefficients using a consistency definition. The between-measure variance is excluded from the denominator variance.

c. This estimate is computed assuming the interaction effect is absent, because it is not estimable otherwise.

DSB_RMSSD_ABS

Scale: ALL VARIABLES

Case Processing Summary

		N	%
Cases	Valid	30	100.0
	Excluded[a]	0	.0
	Total	30	100.0

a. Listwise deletion based on all variables in the procedure.

Reliability Statistics

Cronbach's Alpha	N of Items
.996	2

Intraclass Correlation Coefficient

	Intraclass Correlation[b]	95% Confidence Interval		F Test with True Value 0		
		Lower Bound	Upper Bound	Value	df1	df2
Single Measures	.992[a]	.983	.996	234.813	29	29
Average Measures	.996[c]	.991	.998	234.813	29	29

Intraclass Correlation Coefficient

	F Test with ..
	Sig
Single Measures	<.001
Average Measures	<.001

Two-way mixed effects model where people effects are random and measures effects are fixed.

a. The estimator is the same, whether the interaction effect is present or not.

b. Type A intraclass correlation coefficients using an absolute agreement definition.

c. This estimate is computed assuming the interaction effect is absent, because it is not estimable otherwise.

DSB_HFNU_CONS

Scale: ALL VARIABLES

Case Processing Summary

		N	%
Cases	Valid	30	100.0
	Excluded[a]	0	.0
	Total	30	100.0

a. Listwise deletion based on all variables in the procedure.

Reliability Statistics

Cronbach's Alpha	N of Items
.974	2

Intraclass Correlation Coefficient

	Intraclass Correlation[b]	95% Confidence Interval		F Test with True Value 0			
		Lower Bound	Upper Bound	Value	df1	df2	Sig
Single Measures	.948[a]	.895	.975	37.750	29	29	<.001
Average Measures	.974[c]	.944	.987	37.750	29	29	<.001

Two-way mixed effects model where people effects are random and measures effects are fixed.

a. The estimator is the same, whether the interaction effect is present or not.

b. Type C intraclass correlation coefficients using a consistency definition. The between-measure variance is excluded from the denominator variance.

c. This estimate is computed assuming the interaction effect is absent, because it is not estimable otherwise.

DSB_HFNU_ABS

Scale: ALL VARIABLES

Case Processing Summary

		N	%
Cases	Valid	30	100.0
	Excluded[a]	0	.0
	Total	30	100.0

a. Listwise deletion based on all variables in the procedure.

Reliability Statistics

Cronbach's Alpha	N of Items
.974	2

Intraclass Correlation Coefficient

	Intraclass Correlation[b]	95% Confidence Interval		F Test with True Value 0		
		Lower Bound	Upper Bound	Value	df1	df2
Single Measures	.938[a]	.849	.972	37.750	29	29
Average Measures	.968[c]	.918	.986	37.750	29	29

Intraclass Correlation Coefficient

	F Test with ..
	Sig
Single Measures	<.001
Average Measures	<.001

Two-way mixed effects model where people effects are random and measures effects are fixed.

a. The estimator is the same, whether the interaction effect is present or not.

b. Type A intraclass correlation coefficients using an absolute agreement definition.

c. This estimate is computed assuming the interaction effect is absent, because it is not estimable otherwise.

Norm_RMSSD_CONS

Scale: ALL VARIABLES

Case Processing Summary

		N	%
Cases	Valid	29	100.0
	Excluded[a]	0	.0
	Total	29	100.0

a. Listwise deletion based on all variables in the procedure.

Reliability Statistics

Cronbach's Alpha	N of Items
.988	2

Intraclass Correlation Coefficient

	Intraclass Correlation[b]	95% Confidence Interval		F Test with True Value 0		
		Lower Bound	Upper Bound	Value	df1	df2
Single Measures	.976[a]	.949	.988	80.668	28	28
Average Measures	.988[c]	.974	.994	80.668	28	28

Intraclass Correlation Coefficient

	F Test with ..
	Sig
Single Measures	<.001
Average Measures	<.001

Two-way mixed effects model where people effects are random and measures effects are fixed.

a. The estimator is the same, whether the interaction effect is present or not.

b. Type C intraclass correlation coefficients using a consistency definition. The between-measure variance is excluded from the denominator variance.

c. This estimate is computed assuming the interaction effect is absent, because it is not estimable otherwise.

Norm_RMSSD_ABS

Scale: ALL VARIABLES

Case Processing Summary

		N	%
Cases	Valid	29	100.0
	Excluded[a]	0	.0
	Total	29	100.0

a. Listwise deletion based on all variables in the procedure.

Reliability Statistics

Cronbach's Alpha	N of Items
.988	2

Intraclass Correlation Coefficient

	Intraclass Correlation[b]	95% Confidence Interval		F Test with True Value 0			
		Lower Bound	Upper Bound	Value	df1	df2	Sig
Single Measures	.976[a]	.950	.989	80.668	28	28	<.001
Average Measures	.988[c]	.974	.994	80.668	28	28	<.001

Two-way mixed effects model where people effects are random and measures effects are fixed.

a. The estimator is the same, whether the interaction effect is present or not.

b. Type A intraclass correlation coefficients using an absolute agreement definition.

c. This estimate is computed assuming the interaction effect is absent, because it is not estimable otherwise.

Norm_HFNU_CONS

Scale: ALL VARIABLES

Case Processing Summary

		N	%
Cases	Valid	29	100.0
	Excluded[a]	0	.0
	Total	29	100.0

a. Listwise deletion based on all variables in the procedure.

Reliability Statistics

Cronbach's Alpha	N of Items
.980	2

Intraclass Correlation Coefficient

	Intraclass Correlation[b]	95% Confidence Interval		F Test with True Value 0			
		Lower Bound	Upper Bound	Value	df1	df2	Sig
Single Measures	.961[a]	.920	.982	50.910	28	28	<.001
Average Measures	.980[c]	.958	.991	50.910	28	28	<.001

Two-way mixed effects model where people effects are random and measures effects are fixed.

a. The estimator is the same, whether the interaction effect is present or not.

b. Type C intraclass correlation coefficients using a consistency definition. The between-measure variance is excluded from the denominator variance.

c. This estimate is computed assuming the interaction effect is absent, because it is not estimable otherwise.

Norm_HFNU_ABS

Scale: ALL VARIABLES

Case Processing Summary

		N	%
Cases	Valid	29	100.0
	Excluded[a]	0	.0
	Total	29	100.0

a. Listwise deletion based on all variables in the procedure.

Reliability Statistics

Cronbach's Alpha	N of Items
.980	2

Intraclass Correlation Coefficient

	Intraclass Correlation[b]	95% Confidence Interval		F Test with True Value 0		
		Lower Bound	Upper Bound	Value	df1	df2
Single Measures	.962[a]	.922	.982	50.910	28	28
Average Measures	.981[c]	.959	.991	50.910	28	28

Intraclass Correlation Coefficient

	F Test with ..
	Sig
Single Measures	<.001
Average Measures	<.001

Two-way mixed effects model where people effects are random and measures effects are fixed.

a. The estimator is the same, whether the interaction effect is present or not.

b. Type A intraclass correlation coefficients using an absolute agreement definition.

c. This estimate is computed assuming the interaction effect is absent, because it is not estimable otherwise.
