# Supplementary material for: Evaluation of In-Ear and Fingertip-Based Photoplethysmography Sensors for Measuring Cardiac Vagal Tone Relevant Heart Rate Variability Parameters
Source: Sensors (Basel). 2025 Feb 28;25(5):1485. doi: 10.3390/s25051485 (PMC11902391; doi:10.3390/s25051485)
Supplement: Supplementary file 1 [file sensors-25-01485-s001.zip › PDFS2.pdf]

DSB_RMSSD_CONS

Scale: ALL VARIABLES

Case Processing Summary

		N	%
Cases	Valid	27	100.0
	Excluded[a]	0	.0
	Total	27	100.0

a. Listwise deletion based on all variables in the procedure.

Reliability Statistics

Cronbach's Alpha	N of Items
.975	2

Intraclass Correlation Coefficient

	Intraclass Correlation[b]	95% Confidence Interval		F Test with True Value 0		
		Lower Bound	Upper Bound	Value	df1	df2
Single Measures	.951[a]	.896	.978	40.070	26	26
Average Measures	.975[c]	.945	.989	40.070	26	26

Intraclass Correlation Coefficient

	F Test with ..
	Sig
Single Measures	<.001
Average Measures	<.001

Two-way mixed effects model where people effects are random and measures effects are fixed.

a. The estimator is the same, whether the interaction effect is present or not.

b. Type C intraclass correlation coefficients using a consistency definition. The between-measure variance is excluded from the denominator variance.

c. This estimate is computed assuming the interaction effect is absent, because it is not estimable otherwise.

DSB_RMSSD_ABS

Scale: ALL VARIABLES

Case Processing Summary

		N	%
Cases	Valid	27	100.0
	Excluded[a]	0	.0
	Total	27	100.0

a. Listwise deletion based on all variables in the procedure.

Reliability Statistics

Cronbach's Alpha	N of Items
.975	2

Intraclass Correlation Coefficient

	Intraclass Correlation[b]	95% Confidence Interval		F Test with True Value 0		
		Lower Bound	Upper Bound	Value	df1	df2
Single Measures	.951[a]	.895	.977	40.070	26	26
Average Measures	.975[c]	.945	.988	40.070	26	26

Intraclass Correlation Coefficient

	F Test with ..
	Sig
Single Measures	<.001
Average Measures	<.001

Two-way mixed effects model where people effects are random and measures effects are fixed.

a. The estimator is the same, whether the interaction effect is present or not.

b. Type A intraclass correlation coefficients using an absolute agreement definition.

c. This estimate is computed assuming the interaction effect is absent, because it is not estimable otherwise.

DSB_HFNU_CONS

Scale: ALL VARIABLES

Case Processing Summary

		N	%
Cases	Valid	27	100.0
	Excluded[a]	0	.0
	Total	27	100.0

a. Listwise deletion based on all variables in the procedure.

Reliability Statistics

Cronbach's Alpha	N of Items
.733	2

Intraclass Correlation Coefficient

	Intraclass Correlation[b]	95% Confidence Interval		F Test with True Value 0		
		Lower Bound	Upper Bound	Value	df1	df2
Single Measures	.579[a]	.261	.783	3.746	26	26
Average Measures	.733[c]	.414	.878	3.746	26	26

Intraclass Correlation Coefficient

	F Test with ..
	Sig
Single Measures	<.001
Average Measures	<.001

Two-way mixed effects model where people effects are random and measures effects are fixed.

a. The estimator is the same, whether the interaction effect is present or not.

b. Type C intraclass correlation coefficients using a consistency definition. The between-measure variance is excluded from the denominator variance.

c. This estimate is computed assuming the interaction effect is absent, because it is not estimable otherwise.

DSB_HFNU_ABS

Scale: ALL VARIABLES

Case Processing Summary

		N	%
Cases	Valid	27	100.0
	Excluded[a]	0	.0
	Total	27	100.0

a. Listwise deletion based on all variables in the procedure.

Reliability Statistics

Cronbach's Alpha	N of Items
.733	2

Intraclass Correlation Coefficient

	Intraclass Correlation[b]	95% Confidence Interval		F Test with True Value 0		
		Lower Bound	Upper Bound	Value	df1	df2
Single Measures	.538[a]	.204	.759	3.746	26	26
Average Measures	.699[c]	.339	.863	3.746	26	26

Intraclass Correlation Coefficient

	F Test with ..
	Sig
Single Measures	<.001
Average Measures	<.001

Two-way mixed effects model where people effects are random and measures effects are fixed.

a. The estimator is the same, whether the interaction effect is present or not.

b. Type A intraclass correlation coefficients using an absolute agreement definition.

c. This estimate is computed assuming the interaction effect is absent, because it is not estimable otherwise.

Norm_RMSSD_CONS

Scale: ALL VARIABLES

Case Processing Summary

		N	%
Cases	Valid	28	100.0
	Excluded[a]	0	.0
	Total	28	100.0

a. Listwise deletion based on all variables in the procedure.

Reliability Statistics

Cronbach's Alpha	N of Items
.960	2

Intraclass Correlation Coefficient

	Intraclass Correlation[b]	95% Confidence Interval		F Test with True Value 0		
		Lower Bound	Upper Bound	Value	df1	df2
Single Measures	.924[a]	.842	.964	25.257	27	27
Average Measures	.960[c]	.914	.982	25.257	27	27

Intraclass Correlation Coefficient

	F Test with ..
	Sig
Single Measures	<.001
Average Measures	<.001

Two-way mixed effects model where people effects are random and measures effects are fixed.

a. The estimator is the same, whether the interaction effect is present or not.

b. Type C intraclass correlation coefficients using a consistency definition. The between-measure variance is excluded from the denominator variance.

c. This estimate is computed assuming the interaction effect is absent, because it is not estimable otherwise.

Norm_RMSSD_ABS

Scale: ALL VARIABLES

Case Processing Summary

		N	%
Cases	Valid	28	100.0
	Excluded[a]	0	.0
	Total	28	100.0

a. Listwise deletion based on all variables in the procedure.

Reliability Statistics

Cronbach's Alpha	N of Items
.960	2

Intraclass Correlation Coefficient

	Intraclass Correlation[b]	95% Confidence Interval		F Test with True Value 0		
		Lower Bound	Upper Bound	Value	df1	df2
Single Measures	.906[a]	.763	.960	25.257	27	27
Average Measures	.951[c]	.866	.979	25.257	27	27

Intraclass Correlation Coefficient

	F Test with ..
	Sig
Single Measures	<.001
Average Measures	<.001

Two-way mixed effects model where people effects are random and measures effects are fixed.

a. The estimator is the same, whether the interaction effect is present or not.

b. Type A intraclass correlation coefficients using an absolute agreement definition.

c. This estimate is computed assuming the interaction effect is absent, because it is not estimable otherwise.

Norm_HFNU_CONS

Scale: ALL VARIABLES

Case Processing Summary

		N	%
Cases	Valid	28	100.0
	Excluded[a]	0	.0
	Total	28	100.0

a. Listwise deletion based on all variables in the procedure.

Reliability Statistics

Cronbach's Alpha	N of Items
.915	2

Intraclass Correlation Coefficient

	Intraclass Correlation[b]	95% Confidence Interval		F Test with True Value 0			F Test with ..
		Lower Bound	Upper Bound	Value	df1	df2	Sig
Single Measures	.844[a]	.691	.925	11.822	27	27	<.001
Average Measures	.915[c]	.817	.961	11.822	27	27	<.001

Two-way mixed effects model where people effects are random and measures effects are fixed.

a. The estimator is the same, whether the interaction effect is present or not.

b. Type C intraclass correlation coefficients using a consistency definition. The between-measure variance is excluded from the denominator variance.

c. This estimate is computed assuming the interaction effect is absent, because it is not estimable otherwise.

Norm_HFNU_ABS

Scale: ALL VARIABLES

Case Processing Summary

		N	%
Cases	Valid	28	100.0
	Excluded[a]	0	.0
	Total	28	100.0

a. Listwise deletion based on all variables in the procedure.

Reliability Statistics

Cronbach's Alpha	N of Items
.915	2

Intraclass Correlation Coefficient

	Intraclass Correlation[b]	95% Confidence Interval		F Test with True Value 0		
		Lower Bound	Upper Bound	Value	df1	df2
Single Measures	.818[a]	.606	.916	11.822	27	27
Average Measures	.900[c]	.755	.956	11.822	27	27

Intraclass Correlation Coefficient

	F Test with ..
	Sig
Single Measures	<.001
Average Measures	<.001

Two-way mixed effects model where people effects are random and measures effects are fixed.

a. The estimator is the same, whether the interaction effect is present or not.

b. Type A intraclass correlation coefficients using an absolute agreement definition.

c. This estimate is computed assuming the interaction effect is absent, because it is not estimable otherwise.
